# Optical properties of Ag nanoparticle-polymer composite film based on two-dimensional Au nanoparticle array film

**DOI:** 10.1186/1556-276X-9-155

**Published:** 2014-03-31

**Authors:** Long-De Wang, Tong Zhang, Xiao-Yang Zhang, Yuan-Jun Song, Ruo-Zhou Li, Sheng-Qing Zhu

**Affiliations:** 1 Key Laboratory of Micro-Inertial Instrument and Advanced Navigation Technology, Ministry of Education, and School of Electronic Science and Engineering, Southeast University, Nanjing 210096, People's Republic of China; 2 Department of Chemistry and Chemical Engineering, Huainan Normal University, Huainan 232001, People's Republic of China; 3 Suzhou Key Laboratory of Metal Nano-Optoelectronic Technology, Suzhou Research Institute of Southeast University, Suzhou 215123, People's Republic of China

**Keywords:** Metal nanocomposite, Optical properties, Localized surface plasmon resonance, Surface plasmon-enhanced fluorescence

## Abstract

The nanocomposite polyvinyl pyrrolidone (PVP) films containing Ag nanoparticles and Rhodamine 6G are prepared on the two-dimensional distinctive continuous ultrathin gold nanofilms. We investigate the optical properties and the fluorescence properties of silver nanoparticles-PVP polymer composite films influenced by Ag nanoparticles and Au nanoparticles. Absorption spectral analysis suggests that the prominently light absorption in Ag nanowire/PVP and Ag nanowire/PVP/Au film arises from the localized surface plasmon resonance of Ag nanowire and Au nanofilm. The enhanced fluorescence is observed in the presence of Ag nanowire and Au nanofilm, which is attributed to the excitation of surface plasmon polariton resonance of Ag nanowire and Au nanofilm. The gold nanofilm is proven to be very effective fluorescence resonance energy transfer donors. The fabricated novel structure, gold ultrathin continuous nanofilm, possesses high surface plasmon resonance properties and prominent fluorescence enhancement effect. Therefore, the ultrathin continuous gold nanofilm is an active substrate on nanoparticle-enhanced fluorescence.

## Background

Noble metal nanoparticles with strong localized surface plasmon resonances (LSPRs) have attracted great interests in fields such as nanoscale photonics, biological sensing, surface-enhanced Raman scattering (SERS), photocatalytic and photoelectron-chemical [1], plasmonic absorption enhancement of solar cell [2-10], nonlinear optics [11-14], and plasmon-enhanced fluorescence [15-22]. Localized plasmons are the collective oscillations of free electrons in metal nanoparticles. The LSPRs arising from the excitation of a collective electron oscillation within the metallic nanostructure induced by the incident light lead to enormous optical local-field enhancement and a dramatic wavelength selective photon scattering at the nanoscale [23-26].

Nanocomposites consisting of metal nanoparticles dispersed in a matrix of insulating materials such as polymers, ceramics, or glasses have recently received increased interest as advanced technological materials because of their unique physical properties. The optical properties of noble metal nanoparticles and their application in surface-enhanced photoluminescence are hot in the study of nanoscience. Recently, investigations of the surface enhancement effect on of the fluophor fluorescence have opened up a new methodology for modulating and improving optical properties. The effects of Ag nanoparticles on fluorescence properties of the dye molecules such as Rhodamine B and Nile blue were reported and observed for strong coupling of the particle plasmon resonance to the molecules. Rhodamine (R6G) is frequently used as one of the most efficient laser dyes characterized by a high-efficiency fluorescence band around 560 nm. The fluorescence properties of R6G have been a subject of great interest because of their potential applications as optical signal amplification and light-emitting diode.

We have recently reported a novel structure gold ultrathin continuous nanofilm possessing high surface plasmon resonance properties and boasting a high SERS enhancement factor [27,28]. As a continual effort, here we report the composite films of silver nanowire, nanosphere, and R6G-doped polyvinyl pyrrolidone (PVP) polymer on gold nanocrystal deposited on glass substrate. We research the linear absorption and surface plasmon-enhanced fluorescence optical properties of Ag nanoparticles-polymer composite film. Our results suggest that the ultrathin continuous gold nanofilm can obviously enhance fluorescence optical properties. The interactions of the light and metal composite nanostructures generate new phenomena and realize a new function, which has potential applications in the nanooptics field.

## Methods

### The fabrication of continuous ultrathin gold nanofilm

Our approach is based on the formation of Au nanofilms on glass utilizing magnetron sputtering deposition of metal atoms. The glass substrate was first cleaned with detergent then ultrasonicated in acetone and isopropyl alcohol for further cleaning and subsequently dried in a vacuum oven at 80°C for 3 h. Metallic gold is sputtered on glass using magnetron sputtering in electrical current 0.38 A, vacuum 0.15 Pa, and Ar flux 25 sccm, discharging at 1 s.

### Chemical synthesis of silver nanowires and nanospheres

We used a colloidal synthesis method to prepare silver nanowires improved from literature [29]. At room temperature, l mL ethylene glycol (EG) solution with silver nitrate (AgNO_3_) (0.9 M) and 0.6 mL EG solution with sodium chloride (NaCl) (0.01 M) were added into 18.4 mL EG solution of PVP (MW = 1,300,000) (2.7 M in terms of the repeating unit). Then the mixture was refluxed 185°C for 20 min. After the above processes, the excess PVP and EG were removed by adding deionized water centrifuging at 14,000 rpm for 10 min for three times. The centrifugation ensures that all the products can be collected for the sake of statistics of shapes and size.

In a typical synthesis of quasi-spherical nanoparticles, 0.05 g of AgNO_3_ and 0.20 g of PVP were dissolved in 20 mL of EG at room temperature. The solution was then heated at 160°C in an oil bath for 1.5 h.

### The preparation of silver nanoparticle-PVP polymer composite film

The certain concentration of EG colloidal solutions of silver nanowires, silver nanospheres, R6G, and PVP was dip-coated on glass or gold nanofilm, respectively. The silver nanoparticle-polymer composite films were baked at 60°C for 36 h in a vacuum oven for the complete removal of the solvent EG from the films, which is very important to form a good film.

### The UV-vis-NIR absorption spectra and fluorescence spectra measurements

The UV-vis-NIR absorption spectra were recorded with a fiber-optic spectrometer (PG2000). Fluorescence spectra were registered with a Shimadzu RF-5301PC spectrofluorophotometer (Shimadzu Corp., Kyoto, Japan).

## Results and discussion

### Morphology of fabricated gold nanofilms

**Figure 1:**
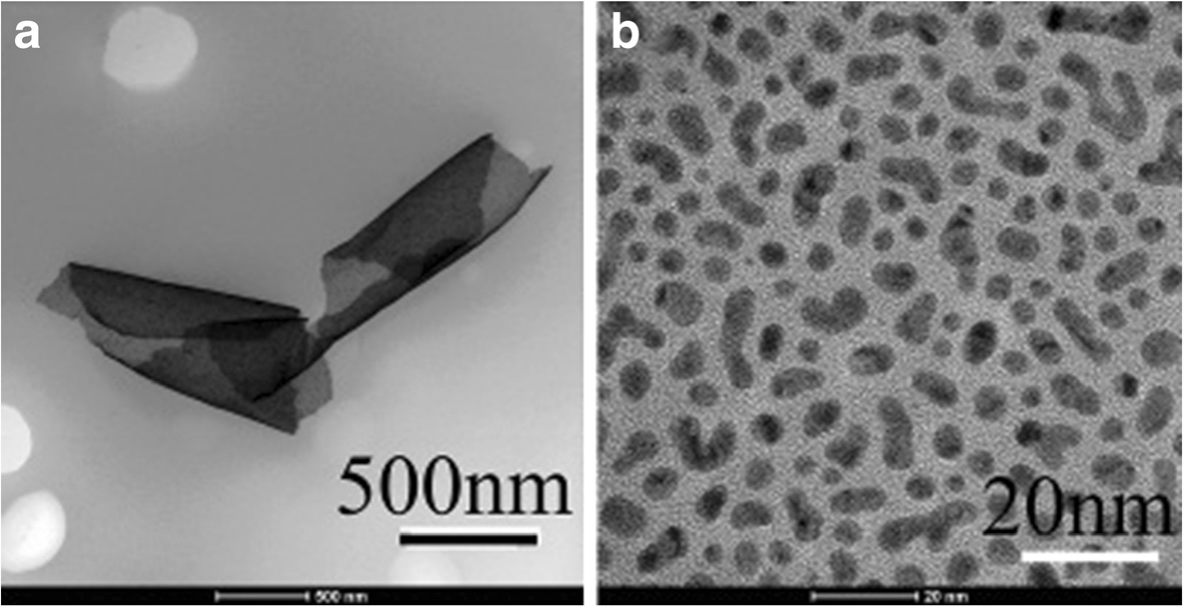
**The TEM micrographs of the obtained gold continuous nanofilms. (a)** The folded nanofilm. **(b)** The structure of the continuous nanofilm.

Figure 1 shows the morphology of fabricated continuous ultrathin gold nanofilms. From Figure 1a the folded nanofilm can be clearly seen as continuous and flexible. From Figure 1b we know that the nanofilm is composed of randomly distributed gold nanoparticles with uniform-sized steady link and ultrathin structure. Within the film the size of the gold nanoparticles is only about 10 nm. The distance between nanoparticles is in sub-10 nm which was filled with even thinner amorphous gold, which can be observed from the high-resolution transmission electron microscopy (TEM) image shown in Figure 1b.

### SEM micrographs of the silver nanowire and nanosphere

**Figure 2:**
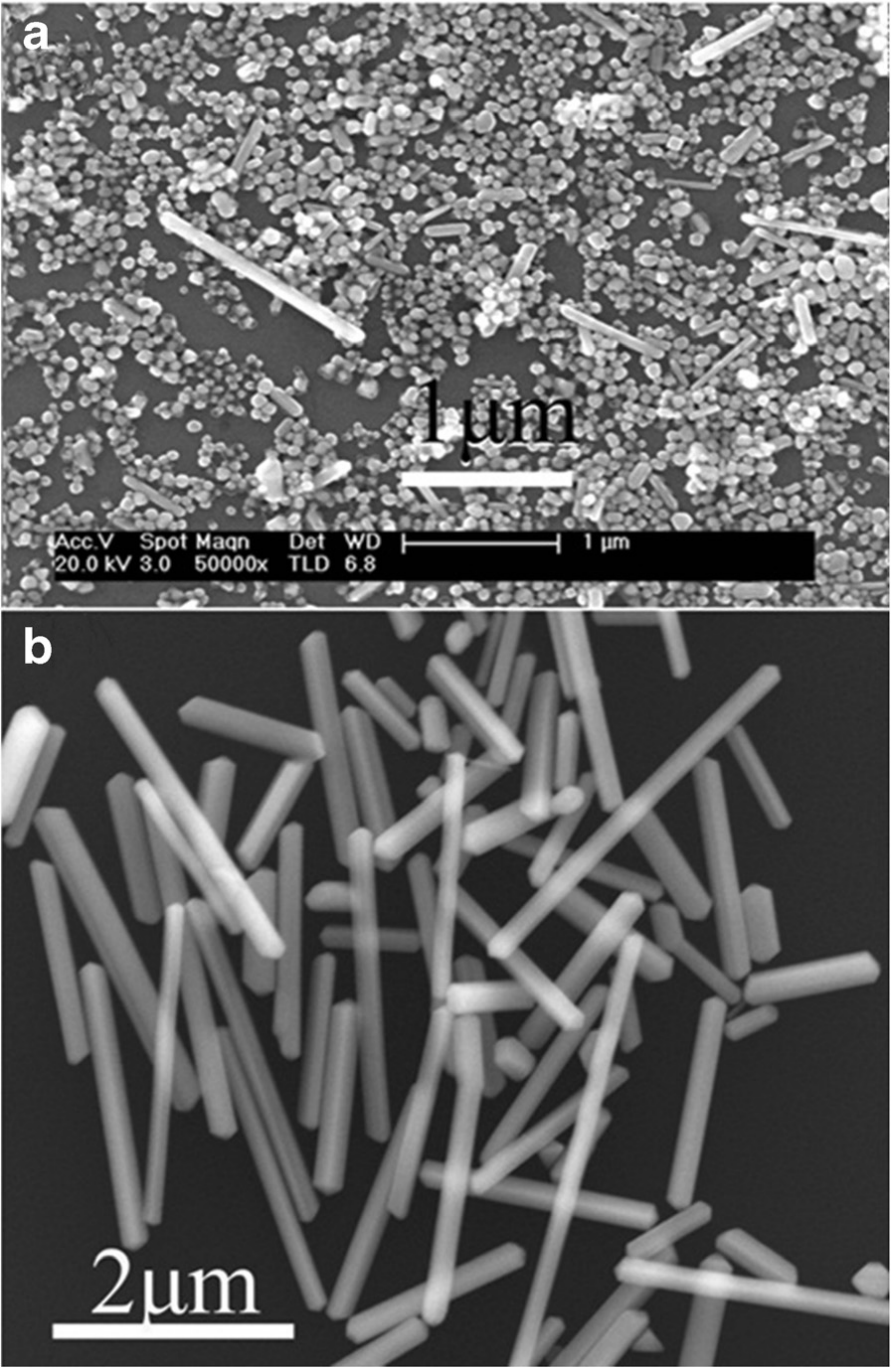
SEM micrographs of the synthesized silver (a) nanosphere and (b) nanowire.

Figure 2 shows a series of silver nanocrystals prepared in the presence of PVP. The scanning electron microscopy (SEM) image in Figure 2a indicates the silver nanospheres with uniform size around 60 nm apart from a small portion of the nanowires. The morphologies of silver nanowires in Figure 2b show the nanowires with different aspect ratios, and the nanowires have very broad size distribution. The length of synthesized longest silver nanowire is about 4 μm.

### UV-vis absorption spectra of the nanoparticle-polymer composite film on the Au nanofilm

**Figure 3:**
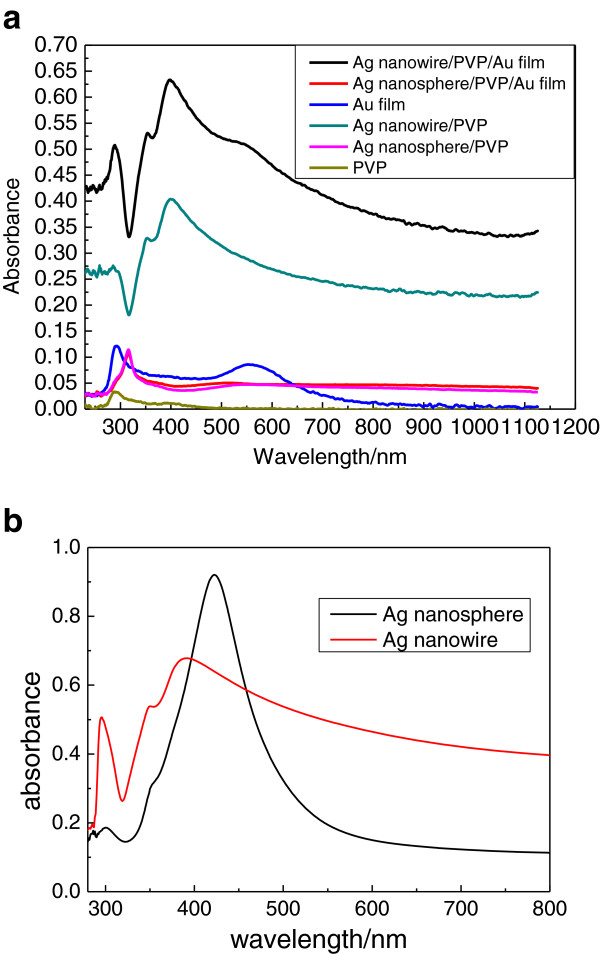
**The UV-vis absorption spectra. (a)** Silver nanoparticle-polymer composite film based on two-dimensional Au nanoparticle array film. **(b)** Silver nanoparticle solution.

Figure 3a shows the comparison of the optical absorption spectra of Ag nanosphere/PVP, Ag nanowire/PVP, Ag nanosphere/PVP/Au film, and Ag nanowire/PVP/Au film. Figure 3b shows the optical absorption spectra of Ag nanoparticles solution. The resonance bands of the plasmonic nanocrystals are mainly dependent on the distribution of the electromagnetic field on the surface of the metal nanocrystals. The absorption of the Ag nanowire/PVP film comes from the surface plasmon resonance of Ag nanowire. Compared to Ag nanowire/PVP, the intensity and the peak position of the absorption band of Ag nanowire/PVP/Au film in Figure 3a have more strength and a little red shift, respectively. These are contributed from the coupling resonant excitation of surface plasmon polaritons of Ag nanowire and near-surface plasmon polaritons of Au nanoparticles on the ultrathin Au film. The absorption peak at 560 nm of ultrathin gold film is also observed on the Ag nanowire/PVP/Au film. The peak of 370 nm ascribes to the localized surface plasmon resonance effect of silver nanowires. The gold nanofilm observably enhances absorbance of silver nanowires. The absorbance of Ag nanowire is apparently higher than that of Ag nanosphere. Under the action of gold nanofilm, the absorbance of Ag nanowire/PVP/Au film is the highest, which can be ascribed to the surface plasmon resonance absorption of Ag nanowire and Au nanoparticles.

However, the absorbances of Ag nanosphere/PVP and Ag nanosphere/PVP/Au film are very weak. In addition, the absorbance resonance peak of silver nanospheres has obviously blueshifted. Meanwhile, the absorption peak at 560 nm of ultrathin gold film disappeared in the Ag nanosphere/PVP/Au film, which means that the surface plasma resonance (SPR) peak of Ag nanosphere is not consistent with that of the Au nanofilm. Compared to Ag nanosphere, the longer Ag nanowire has sharper plasmon resonance that leads to red-shifted plasmon resonance and ensures a better overlap between plasmon resonance and absorption band of Au nanofilm. So there is no resonance-enhanced absorption between the Ag nanosphere and Au nanofilm. It is an important point to keep in mind that the SPR wavelength and the resonance intensity is greatly influenced by the kind of metal, particle size and shape, aggregation condition of particles, and so on.

### The fluorescence optical properties of nanoparticle-polymer composite film on the surface of the Au nanofilm/glass

**Figure 4:**
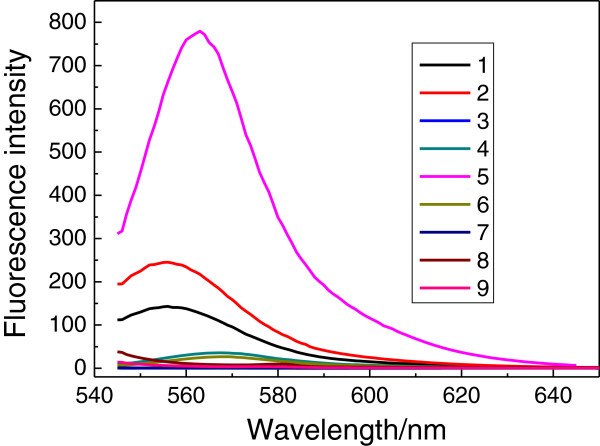
**Fluorescence spectra.** 1 R6G/PVP. 2 R6G /PVP/Au film. 3 R6G/Ag nanowire/PVP. 4 R6G/Ag nanosphere/PVP. 5 R6G/Ag nanowire/PVP/Au film. 6 R6G/Ag nanosphere/PVP/Au film. 7 Ag nanosphere/PVP. 8 PVP. 9 Ag nanowire/PVP films.

The effects of the existence of Ag nanoparticles and Au nanofilm on the fluorescence from the R6G/PVP films are further investigated, as shown in Figure 4. There is no fluorescence from the R6G/Ag nanowire/PVP, R6G/Ag nanosphere/PVP, R6G/Ag nanosphere/PVP/Au film, Ag nanosphere/PVP, and Ag nanowire/PVP films, according to in Figure 4. Thus, the fluorescence peaks of 563 nm shown in Figure 4 are attributed to electric transition of π-π* of R6G doped in the PVP films. The enhanced fluorescence is observed in the R6G/Ag nanowire/PVP/Au film and R6G/PVP/Au film, and the enhanced factor (*I*_c_/*I*_b_) is about 7.7 and 2.3, respectively. The *I*_c_ is the fluorescence absorption peaks of R6G/Ag nanowire/PVP/Au film and R6G/PVP/Au film at 560 nm nearby, respectively. The *I*_b_ is the fluorescence absorption peak of R6G/PVP at 560 nm nearby.

The fluorescence quenching in the metal colloid film has been observed in the R6G/Ag nanowire/PVP, R6G/Ag nanosphere/PVP, R6G/Ag nanosphere/PVP/Au film, according to Figure 4. The SPR resonance absorption peak at 560 nm of Au nanoparticle is consistent with the R6G absorption peak, therefore, the enhanced fluorescence is observed in the R6G/PVP/Au film. According to the optical absorption spectrum of Ag nanowire/PVP/Au film in Figure 3, there is strong optical absorption at 563 nm nearby. Therefore, the obviously enhanced fluorescence is observed in the R6G/Ag nanowire/PVP/Au film. These phenomena are ascribed to surface-enhanced fluorescence, resulting from surface plasmon resonance of Ag nanowire and Au nanoparticle. Especially, the Ag nanowires and Au nanoparticles possess the capacity to induce strongly enhanced fluorescence due to the coupling resonance of surface plasmon polaritons of Ag nanowire and Au nanoparticle. For surface-enhanced fluorescence it is very important that R6G should be closed to the surface of Ag nanoparticles, this is realized under the help of PVP. However, fluorescence quenching occurred once R6G's immediate contact with the metal nanoparticles results in nonradiative energy transfer between the R6G and metal nanoparticles [30].

Without the strong resonance absorption at 560 nm nearby of the Ag nanosphere and the Au nanofilm, there is no fluorescence from the R6G/Ag nanosphere/PVP and R6G/Ag nanosphere/PVP/Au film. Even though the Ag nanowire/PVP has optical absorption at 560 nm nearby in Figure 3, no fluorescence in R6G/Ag nanowire/PVP is observed without Au nanofilm. Hereby, it is the Au nanofilm that possesses the surface plasmon-enhanced fluorescence. The gold nanofilm is proven to be very effective fluorescence resonance energy transfer donors. The main factors that affect surface plasmon-enhanced fluorescence are (1) nanoparticle size and shape of the metal; (2) the distance between metal nanoparticles and luminophor; and (3) the electromagnetic field effect in exciting light, surface plasmon polaritons, and fluorescence of luminophor.

## Conclusions

The absorption and fluorescence spectra of the nanocomposite PVP films with Ag nanoparticles and Rhodamine 6G prepared on the two-dimensional continuous ultrathin gold nanofilm have been studied. Absorption spectral analysis suggests that the prominently light absorption in Ag nanowire/PVP and Ag nanowire/PVP/Au film arises from the localized surface plasmons resonance of Ag nanowire and Au nanofilm. The enhanced fluorescence is observed in the presence of Ag nanowire and gold nanofilm, which is attributed to the excitation of surface plasmon polaritons resonance of Ag nanowire and gold nanofilm. We have produced a two-dimensional continuous ultrathin gold nanofilm which possesses high local-field enhancement effect, high SERS activity, and surface-enhanced fluorescence.

## Competing interests

The authors declare that they have no competing interests.

## Authors’ contributions

L-DW carried out the design and prepared the nanocomposite film, performed the optical absorption and fluorescence analysis of nanocomposite film, and drafted the manuscript. R-ZL participated in the fabrication of gold films. X-YZ participated in the absorption spectra measurement. Y-JS participated in the synthesis of silver nanoparticles. TZ and S-QZ read the manuscript and contributed to its improvement. All authors read and approved the final manuscript.
